# Supplementary material for: Blended and collaborative learning: Case of a multicultural graduate classroom in Taiwan
Source: PLoS One. 2022 Apr 28;17(4):e0267692. doi: 10.1371/journal.pone.0267692 (PMC9049308; doi:10.1371/journal.pone.0267692)
Supplement: S1 File — (DOC) [file pone.0267692.s002.doc]

***Community of Inquiry Survey Instrument****

***Teaching Presence***

*Design & Organization*

1. The instructor clearly communicated important course topics.

2. The instructor clearly communicated important course goals.

3. The instructor provided clear instructions on how to participate in course learning activities.

4. The instructor clearly communicated important due dates/time frames for learning activities.

*Facilitation*

5. The instructor was helpful in identifying areas of agreement and disagreement on course topics that helped me to learn.

6. The instructor was helpful in guiding the class towards understanding course topics in a way that helped me clarify my thinking.

7. The instructor helped to keep course participants engaged and participating in productive dialogue.

8. The instructor helped keep the course participants on task in a way that helped me to learn.

9. The instructor encouraged course participants to explore new concepts in this course.

10. Instructor actions reinforced the development of a sense of community among course participants.

*Direct Instruction*

11. The instructor helped to focus discussion on relevant issues in a way that helped me to learn.

12. The instructor provided feedback that helped me understand my strengths and weaknesses relative to the course’s goals and objectives.

13. The instructor provided feedback in a timely fashion.

***Social Presence***

*Affective expression*

14. Getting to know other course participants gave me a sense of belonging in the course.

15. I was able to form distinct impressions of some course participants.

16. Online or web-based communication is an excellent medium for social interaction.

*Open communication*

17. I felt comfortable conversing through the online medium.

18. I felt comfortable participating in the course discussions.

19. I felt comfortable interacting with other course participants.

*Group cohesion*

20. I felt comfortable disagreeing with other course participants while still maintaining a sense of trust.

21. I felt that my point of view was acknowledged by other course participants.

22. Online discussions help me to develop a sense of collaboration.

***Cognitive Presence***

*Triggering event*

23. Problems posed increased my interest in course issues.

24. Course activities piqued my curiosity.

25. I felt motivated to explore content related questions.

*Exploration*

26. I utilized a variety of information sources to explore problems posed in this course.

27. Brainstorming and finding relevant information helped me resolve content related questions.

28. Online discussions were valuable in helping me appreciate different perspectives.

*Integration*

29. Combining new information helped me answer questions raised in course activities.

30. Learning activities helped me construct explanations/solutions.

31. Reflection on course content and discussions helped me understand fundamental concepts in this class.

*Resolution*

32. I can describe ways to test and apply the knowledge created in this course.

33. I have developed solutions to course problems that can be applied in practice.

34. I can apply the knowledge created in this course to my work or other non-class related activities.

5 point Likert-type scale

1 = strongly disagree, 2 = disagree, 3 = neutral, 4 = agree, 5 = strongly agree

*Source:

Arbaugh JB, Cleveland-Innes M, Diaz SR, Garrison DR, Ice P, Richardson JC, Swan KP. Developing a community of inquiry instrument: Testing a measure of the community of inquiry framework using a multi-institutional sample. The internet and higher education. 2008 Jan 1;11(3-4):133-6.
