# Supplementary material for: Blended and collaborative learning: Case of a multicultural graduate classroom in Taiwan
Source: PLoS One. 2022 Apr 28;17(4):e0267692. doi: 10.1371/journal.pone.0267692 (PMC9049308; doi:10.1371/journal.pone.0267692)
Supplement: S2 File — (DOCX) [file pone.0267692.s003.docx]

**Categories of Responses**

(*numbers in brackets are identification codes for student respondents*)

**TEACHING PRESENCE**

**Facilitation**

- Teacher try to mix everyone (local and foreign) as much as possible (1&2) Giving of examples to make it clearer – current affairs, her own experience, examples from other cultures, not all according to the book (1); use of Wikipedia and YouTube to explain some things (3)
- Issue of time management, following the rules and not going overtime, do not take too much time on one person (1)
- Teacher very hardworking (1)

**Direct instruction**

- Encouragement of teacher (“feedback in a very peaceful manner; encouragement first before feedback”) – (3)
- Giving of examples to make it clearer – current affairs, her own experience, not all according to the book (1); use of Wikipedia and YouTube to explain some things (3)
- Well, I think the helpful part may be like, because the students may put forward different opinions on a certain problem, then the teacher may do a little summary of the concept or generalization after everyone has finished speaking, and then just It's clearer. (4)
- Consistency of teaching presence online and offline (4)

**SOCIAL PRESENCE**

**Affective expression**

- Grouping is a way to get to know each other personally (1)
- Grouping is done at random (1 & 2)
- Easier to drift, lose attentiveness, forget about other people in online (1&2)

**Open communication**

- Language is a problem during grouping though not very serious (3)
- Face to face with facial expressions, uh... can make you more want to speak. (3)
- In online it will be less able to express (3)
- Personal discomforts: F2F vs online. “It’s not bad to pull, so I feel comfortable”; I actually don’t want to turn on the camera. Because it’s just that we usually don’t talk face to face like this, that is, when we are in class, we don’t look at the faces of nine or ten people at a time, but when online, if everyone turns on the camera, we get a full view. Yes, all people will see, that is, all people will see each other's face very close. (Camera) only open when I speak (1) Open because the teacher wants it (2)
- not really a problem because only seek the face of the teacher, others are ignored (3)

**COGNITIVE PRESENCE**

**Triggering event**

- Impressive is the Q and A where you ask questions and then get to answer another’s questions (1); The most special thing is after Q and A (5)

**Exploration**

- Learning from the Q& A “different people (local and foreign) participate; discuss which question to answer, and then how to answer, and then what you think, what other people think, and then how to present in the end” (1 & 2)

**Others**

- More diverse ways of doing the homework (1); diversity of this type of approach – (3)
- Feeling that the amount of homework is too large, extended indefinitely (Students 1&2)
- Feeling that the time is not enough (2), “Doing class-related activities all week” (extended class hours) (1)
- Discussion of the answers is limited because of the time especially if the particular question needs to be discussed more thoroughly than other types of questions. “Have a very conflicting feeling (because of multitasking)” (2)
- Feeling tired and stressed because of the unpredictability of tasks (1) but there is way around it like giving ample time or reasonable time for completion of home works (1) 🡪 quality of completion is worse (1); unpredictable schedule once or twice but is ok (acceptable; learned to adapt) (3 &10); a little bit heavy because you need to arrange time to do this (3)
- Heavier feeling of mixed than separate. (1) 🡪 if taken with other subjects, it is full. Maybe with only one other subject is better
- Some learning activities time consuming (recording and everybody watches – “It will take longer than I thought. It takes more time than I thought. To make a slideshow, you have to write something similar to a script, and then talk about it, but it may be that I am less proficient in using English. (1)
- Students are very hardworking (1)
- Learning more than the topic itself; metacognition “you can learn in this course, whether it’s innovation-related theories, or learning how to communicate with others, or even simply using language to discuss a topic. You can learn a lot in the process of participating in this class, and you will feel that if this class continues, it will get better and better” (1); effect on communication skills, learn how to be brave and know how to ask and answer questions (3)

**BENEFITS, CHALLENGES, SUGGESTIONS**

- There are some things that cannot be done purely online (2)
- Small breakout rooms in the videoconferencing app would be ideal (1); Online discussion is also possible, but we don’t seem to have any (real) discussion. (3)
- Preference for mixed/blended learning because “because of the learning activities that can be covered, some things that cannot be done online can be done offline, and things that are more inconvenient to perform offline can be completed online.” (1) however Student 2 says “But this will make us have a lot of homework”
- Issue on security of the online teaching tool, Zoom (1) “If you want to mix up, do mixed courses, or promote such things, you still have to pay attention when you use online video teaching software. The information security part”
- Q and A as the most recognizable feature of the class (collaborative work); left most impression to the students as all of them mentioned it
- Teacher as sage on stage and is the authority; even if difficult there is no complaints, have to adjust (East/Asia culture)
- Class is full, acceptable because it is pure social science, humanities not sciences (3)
- Almost the same in terms of comfortable but in the classroom, it is more convenient to discuss (3)
- Technical issues in online (3)
- Video tape so you will not miss anything (3)
- Time gap in online (3)
- Discussion style is troublesome (3)
- Online is difficult (3)
- You can rest online (3)
- Learning effect has been very good (3)

**Main Themes**

1. Central and cohesive role of instructor in BCL Environment (Teaching Presence)

Strong teaching presence and teacher as a cohesive force

1. Collaborative activities: Activating social and cognitive presences
2. Blended learning approach: Rich and full, but time-constrained (Cognitive Presence)

Blended format and varied activities are novel and very rich for students but there is a feeling of being overwhelmed.

1. Conditions
2. Influence of the multicultural aspects (Social Presence)
   1. Holding back
   2. Recognition of teacher authority
