# Supplementary material for: Blended and collaborative learning: Case of a multicultural graduate classroom in Taiwan
Source: PLoS One. 2022 Apr 28;17(4):e0267692. doi: 10.1371/journal.pone.0267692 (PMC9049308; doi:10.1371/journal.pone.0267692)
Supplement: S3 File — (DOCX) [file pone.0267692.s004.docx]

**Interview Details and Questions**

To find out their opinions about the course, the instructor and the students participated in semi-structured interviews, with open-ended questions based mainly on the CoI framework. Each interview lasted between 30-60 minutes per participant, with interview data collected through digital recording then transcribed into a text document.

Below are sample guide questions for the semi-structured interviews:

**For the instructor:**

1. General Questions
2. What made you decide to offer the DOI course using a blended learning format?
3. Would you consider the course to be an on the “run course” or being modified and developed as you go along?
4. Were there certain activities that you do exclusively online, offline? Which can you do in both?
5. What do you think are the opportunities and challenges of blended learning? Would you recommend it to other professors?
6. Teaching Presence:
7. What classroom management issues did you consider when you decided to offer DOI using a blended learning format?
8. Social Presence
9. Please describe how you provide feedback in classroom and in online discussions.
10. How did you encourage social interaction, and open communication in the class (both online and physical)?
11. Cognitive Presence
12. What kind of assigned readings and homework practices did you provide the students in the class?
13. What did you consider as signs or indicators that the students understood the topics/concepts from assigned readings and home works?
14. Do you have any other insights about blended learning that we have not covered?

**For the students:**

These questions are based on the community of inquiry questionnaire.

1. Did you experience blended learning before? If yes, please tell us about your previous experience.
2. In this particular course [BCL course], what are your impressions about the design and organization of the course?
3. Were the following communicated to you clearly by the instructor: course objectives, course schedule, nature of learning activities? Kindly describe how.
4. Can you share how the instructor facilitated the course? Guided you and your classmates to learn/understand the topics?
5. Do you think both online and face to face classed helped you learn/understand the topics in class? Kindly describe in what ways.
6. How did the online or web-based communication help you interact with your classmates?
7. Because this is an EMI course with both local and international students, what can you say about:
   1. Interactions in the class
   2. Collaborative work/sense of collaboration in the class
8. How does the course encourage open communication and comfortable interaction of course participants?
9. Can you describe how the BCL course helped (or did not help) you learn the concepts?
10. What are the types of learning activities that you like/dislike? Were there certain activities that you do exclusively online, offline? Which can you do in both?
