# Supplementary material for: Blended and collaborative learning: Case of a multicultural graduate classroom in Taiwan
Source: PLoS One. 2022 Apr 28;17(4):e0267692. doi: 10.1371/journal.pone.0267692 (PMC9049308; doi:10.1371/journal.pone.0267692)
Supplement: Attachment — Submitted filename: Response to Reviewers_v8.docx [file pone.0267692.s005.docx]

Manuscript PLOS ONE_PONE-D-21-31362

**"Blended and collaborative learning: Case of a multicultural graduate classroom in Taiwan"**

We sincerely appreciate the thoughtful comments of reviewers and have used them to shape the article into what we believe is an improved manuscript. Our responses to the reviewers’ comments are included below:

**REVIEWER #1:**

This study presents a blended and collaborative learning method and discuss its potential as a viable learning approach for graduate classrooms with culturally-diverse students. The topic is interesting. In my view, the findings of this study can provide important insights into instructional practice on blended and collaborative learning. I only have several minor concerns to be addressed.

Comment 1

First, I suggest the authors to highlight more about the importance and significance of blended learning and collaborative learning in the field of educational technology, as evidenced in reviews on educational technology, see the following:

Detecting latent topics and trends in educational technologies over four decades using structural topic modeling: A retrospective of all volumes of Computers & Education

Fifty years of British Journal of Educational Technology: A topic modeling based bibliometric perspective

Response:

Thank you very much for your suggestions. It has clarified even more our understanding of where blended and collaborative learning as a research work stands among the current researchable areas in educational technology. We have highlighted the importance and significance of BCL by adding this paragraph in the manuscript (please see lines 267-325 pages 7-8):

*Studies on blended learning and collaborative learning are among the most cited works, as evidenced in reviews in educational technology; this indicates the significance of BCL as a current and potentially competitive research area. Blended learning, for instance, started receiving increased attention from the academic community since 2003-2006, showing an increasing interest in teaching and learning using technologies and games [39]. The topic of context and collaborative learning is one of the most studied topics, demonstrating its popularity in teaching and learning research, and its importance in promoting the development of various types of knowledge [39].*

Comment 2

It is suggested to include more empirical studies focusing on blended and collaborative learning to highlight the significance of your research. For example,

Kong, S. C., Cheung, W. M. Y., & Zhang, G. (2021). Evaluation of an artificial intelligence literacy course for university students with diverse study backgrounds. Computers and Education: Artificial Intelligence, 100026.

Response:

Thank you for bringing this to our attention. We have included what you recommended in the article. The additional paragraph is found below (please see lines 326-336, page 8):

*Although certain aspects of blended learning and collaborative learning have been previously studied, research describing what actually happens in a blended and collaborative environment and what lessons might be learned from such a study, particularly in a multicultural higher education classroom, is limited. Empirical studies with similar goals and format to the present study have been published before, signaling the importance of reporting the design, implementation, and evaluation of learner-centered educational initiatives in the digital era. An example is a study about an artificial intelligence (AI) literacy course attended by Hong Kong university students who are from diverse backgrounds [40]. The design, implementation, and assessment of the AI course with a flipped learning classroom approach were reported. Quantitative analysis was used to evaluate the course’s impact to students’ conceptual understanding [40].*

Comment 3

Research questions can be more specific, for example, RQ2, what does “knowledge” means?

Response:

Thank you for your comment. For RQ2, we agree that “knowledge” is an imprecise and vague term. We have replaced it with “learnings” which is a type of knowledge that is acquired through study or experience, or a lesson learned from experience (please see lines 501-503, page 11). Please also see below.

*2.What learnings are derived from the implementation of BCL in a multicultural setting in terms of three interdependent CoI elements – teaching, cognitive, and social presences?*

By deciding on the usage of the term “learnings,” we are also consistent with how the findings of the qualitative analysis were presented in the Results section of the paper (page 20)

Comment 4

What are the limitations of this study?

Response:

Thank you for your valuable suggestion. We have added the limitations of the study at the implications and conclusion section (please see lines 2082-2091, page 33). Please see the added paragraph below:

*This study is subject to limitations. First, although the aim of this study was to demonstrate how a BCL approach is integrated in a multicultural classroom and identify the lessons that can be learned from the implementation of BCL, as a single case study, the results may have limited explanatory range and generalizability—a limitation that is usually tied to a broader critique on qualitative methods as a whole. Second, the participants of the course were mostly Asian students; a case of a BCL class with more culturally diverse students—particularly one that includes Western students—can demonstrated stronger representativeness and provide a broader spectrum of cultural differences. Finally, the study has limited quantitative findings to support the claim that BCL is a viable approach for multicultural classrooms; hence, further research is recommended.*

Comment 5

Finally, double-check both definition and usage of acronyms: every acronym should be defined only once (at the first occurrence) and always used afterwards (except for the abstract). There are some mistakes in this aspect. For example, the use of CoI and community of inquiry.

Response:

Thank you for your comment. The acronyms used namely BCL and CoI have been applied consistently in the revised manuscript. The latter has also been reviewed and proofread grammatically.

**REVIEWER #2:**

This paper describes an implementation of a blended and collaborative learning approach in a multicultural graduate course, intending to demonstrate such an implementation can encourage cooperative learning among graduate students of different cultural backgrounds. The paper evaluates the learning outcomes using the Community of Inquiry framework. In general, the paper delivers a teaching and learning event with a theoretical foundation and a well-constructed learning process. Thus, the paper is of interest to the journal and researchers in the relevant field.

The authors can consider the following suggestions:

Comment 1:

1. Organization:

1) It will be better if the authors number the sections and subsections. The current format without numbering may make the audience get lost.

Response:

Thank you for this comment. We have numbered and limited the sections and subsections to three heading levels. We have also clearly demarcated each heading level by using decreasing font size, from top main heading to sub-heading.

2) Line 288. It is confusing to entitle this section as "Results," as it is more about the teaching and learning settings and some basic statistics. The authors can consider separate this section accordingly to make the manuscript logically intact.

Response:

We appreciate this comment, but may we respectfully explain why this section was placed under the Results section: while this section appears, at first glance, to be a documentation of the BCL course, containing a description of the teaching and learning settings, and some basic statistics, it answers our RQ1 (How can BCL be integrated in a multicultural classroom?) by including qualitative findings from the classroom observation analysis. The different parameters, when weaved together, will show how the BCL in a multicultural setting was integrated. We have also revised the 5.1.heading as “The integration of the blended and collaborative learning in a multicultural classroom” to properly address RQ1.

3) Another limitation of the manuscript is that the description of blended and collaborative learning in the current "Results" section is not well-written: the content is too scattered without a good connection, and the writing is not good enough to present a holistic image. The authors are highly suggested modifying this section to make everything straightforward. For example, authors can consider writing a general description to start this section, highlighting the logic and how different subsections are connected.

Response:

Thank you very much for your comment. The description of blended and collaborative learning was numbered accordingly and a general description introducing the different subsections were presented. Originally, we followed all six parameters that constitute the framework for describing the blended learning design. We reduced these parameters to four by grouping related parameters. We also tried to make the description as straightforward as possible. For example, we added a sentence to point out explicitly that this section addresses the first research question. Please see below the general description of the BCL which we added in the first part of the section (please see lines 674-683, page 15):

*In this section, we address the first research question by describing the structure and details of the BCL course following Neumeier’s [9] framework of parameters for the design of blended learning. The framework focuses on specific teaching and learning requirements originally presented under six different parameters; in this study, however, these are combined into four parameters for a more straightforward presentation: (a) mode and integration, or the blended learning approach and nature of activities; (b) teaching method and distribution of learning content; (c) involvement of learning subjects, or the types of interactions taking place; and (d) location, or the physical place where the learning takes place. Together, these parameters were used to describe the BCL design in a multicultural classroom.*

4) The "Results" part does not discuss how this work addresses from a multicultural perspective and impacts the learning of students from minor ethical groups, which is emphasized a lot in the abstract and introduction. Please consider adding discussions regarding the multicultural setting into the manuscript.

Response:

Thank you for this comment to which we agree with fully.

Multicultural perspectives were added to the results part by explaining how the elements of the blended learning design have been put together and implemented in consideration of the local and international students. For example: use of translanguaging, combining locals and foreign students in collaborative activities, use of a broad range of technologies to address communication and mobility issues, etc. Below please find relevant excerpts:

(Lines 736-745, page 16) *“According to the instructor, face-to-face meeting was chosen as the principal mode of the blended learning course to promote more in-person interaction and socialization between local and international students, as management of group dynamics is easier, more flexible, and less structured during in-person sessions. However, the online mode also helped in making the multicultural class function well by presenting opportunities for one-to-one or small-group collaborative learning. According to Gabb [15], managing the group dynamics in a multicultural group of students, centered on measures that determine the psychosocial climate in the classroom, is an important part of a teacher’s repertoire. With the integration of the two modes, the instructor of the BCL course was able to implement measures facilitating interpersonal relationships and meaningful learning.”*

(Lines 787-791, pages 17) “*Translanguaging, or the use of all of one’s linguistic and cognitive resources to teach or learn academic content, was evident during the BCL course. For example, the instructor would use the Mandarin term for the word innovation to make it more understandable to local students and then ask the other international students to give examples of an innovation as they understood it in their own context.”*

(Lines 861-863, pages 18) *“In creating the small groups or pairs, the instructor tried to group together foreign and local students to ensure a “cultural mix;” however, as the local students outnumbered the foreign students, only a few groups had at least one foreign member.”*

Second, multicultural perspectives were added to the four themes describing the learnings derived from the BCL experience. Below are some examples:

(Lines 1231-1236, page 23) *“The students identified the Q&A activity as the “most special thing” about the course. It represented both the collaborative and interactive activity that was recognized by some as a strategy by the instructor to help students learn the topics better; a means to gain different perspectives and feedback and to refine one’s own thinking; and a way of discovering the personalities and backgrounds of classmates through the way they constructed and answered the questions.”*

(Lines 1372-1373, page 25) *“The students attributed the slightly polarized reactions of local and foreign students to the course to language as a multicultural aspect.”*

As to the claim about multicultural setting, more literature can be added to support the hypothesis.

Response:

Thank you for this suggestion. We modified then added another paragraph on the section discussing collaborative learning in multicultural context to support our hypothesis on BCL using the multicultural perspective (please see lines 224-266, pages 6-7).

*Tielman et al. [11] studied the practices and perceptions of both teachers and students in a vocational secondary school in the Netherlands with respect to collaborative learning in a multicultural class. They reported poor collaboration in the multicultural class, as students focused only on their own personal activities. The teachers, too, seemed unaware of their roles in affecting students’ behaviors and the influences of students’ cultural backgrounds in the collaborative process. Le, Janssen, and Wubbels [35] pointed out that students, in general, can encounter difficulties in collaborative learning if they lack collaborative skills such as accepting opposing viewpoints, giving elaborate explanations, providing and receiving help, and negotiating. In a multicultural classroom, where students come from different cultural backgrounds, it could be more challenging to use these collaborative skills. However, Coelho ([36], as cited in [11]) argued that collaborative learning is an appropriate teaching approach in the multicultural context. Gay [37] and Sharan [38] also emphasized the role of teachers in creating a culturally sensitive classroom by being aware of students’ diverse cultural backgrounds and integrating cooperative learning methods [38]. Developing a rich repertoire of multicultural instructional examples when teaching school subjects is important for teachers [37]. Sharan [38] suggested the use of cooperative learning methods and strategies but stressed that only placing students into groups is not sufficient; a partnership needs to be created in a multicultural classroom by the judicious and gradual use of cooperative learning methods with sensitivity to the manifestations of diversity. Moreover, Kumi-Yeboah et al. [28] found that minority graduate students perceived online collaborative learning as providing them with opportunities to share and lead discussions with different perspectives.*

And is there any quantitative results to reveal the effect of the proposed BCL setting on both local students and foreign students? I think it would be interesting if these aspects are discussed.

Response:

Thank you for your comment. There are no direct quantitative results to reveal the effect of the proposed BCL on both the local and foreign students but we have qualitative findings indicating this.

Quantitative data analysis was limited to the CoI survey results reported as mean and SD to each of the three dimensions, teaching presence, and social presence respectively (page 20). Impressions and impacts of the course were elucidated from the interview and classroom observation data. However, we do appreciate this feedback and consider its importance. Hence, we added this issue as a limitation in the Implications and Conclusion section to guide readers on their interpretation of the findings, and to invite/encourage future studies focusing on this (lines 2072-2074, pages 33):

*Finally, the study has limited quantitative findings to support the claim that BCL is a viable approach for multicultural classrooms; hence, further research is recommended.*

Comment 2:

2. Writing:

The writing quality of this paper can be improved. The authors are suggested to make thorough proofreading.

Response:

Thank you very much for this important observation. The typographical errors have been checked and proofread accordingly. We have also removed excess words to make the paper more concise and to enhance its readability.

Typos:

Line 100: "equity" to "equality"

Response:

In this line, we respectfully would like to retain the word “equity.” Though equality and equity are often used interchangeably, we adhere to the definition of “equality” as referring to treatment of everyone in the same exact way, regardless of need or any other individual difference; and “equity,” meaning everyone is provided with what they need to succeed, considering their needs and disadvantages, which is emphasized in the citation (reference [20]). (please see line 174, page 5)

Line 133: "others" to "others' "

Response:

The current form is “others,” referring to people in general. (please see line 180, page 5)

Line 140: "The" to "the"

Response:

We have changed “The” to “the.” (please see line 225, page 6)

Line 298: "exercise" to "exercises"

Response:

Thank you for pointing this out. The term “exercise” had been replaced with the word “course,” as it should aptly be. The whole sentence in the revised manuscript is now “*The participants conducted 15 out of the 18 meetings of the regular class hours in the face-to-face mode, making it the principal mode in this blended learning course, and the remaining 3 meetings in the online mode (synchronous), not including the time spent on collaborative activities such as Q&A, collaborative assignments, and peer evaluation via Google Docs.”* (please see line 691-725, pages 15-16).

and other places.

Response:

We checked the whole paper and found other terms that need correction. We have also asked a language editor to look at our manuscript to ensure that the paper is written in a technically correct manner.

Especially, the paper does not well follow the citation format. For example, multiple in-line citations miss the period sign "." in "et al."

Response:

The multiple-author in-text citations format have been checked and corrected.

As to the use of acronyms, once a short form is defined, please use the acronym throughout the manuscript, like "BCL".

Response:

Thank you for your suggestion. The acronyms used namely BCL and CoI have been applied consistently in the revised manuscript. The latter has also been reviewed and proofread grammatically.

The term "Community of Inquiry" is defined as "CoI", but another form "COI" is used multiple times. Please stick with one form and revise the other one.

Response:

The acronym “CoI” for Community of Inquiry has now been applied consistently in the manuscript. All “COI” have been replaced with “CoI.” The rule of the first mention was also followed.

And "Q and A" may not be a formal usage. Please consider using "Q&A" instead.

Response:

The formal usage has been adopted. All “Q and A” were replaced with “Q&A.”

We greatly appreciate the kind and helpful comments. Following your suggestions/comments (as well as the other reviewer’s comments), we have made significant changes and revisions on the previous version. We hope this current version would be more convincing. Thank you very much.
